# Book Reviews

**Published:** 1822-11

**Authors:** 


### MEDICAL AND SCIENTIFIC BIBLIOGRAPHY.

Vexat censura ...... Corvos.

Art. I.—*Anatomical and Physiological Commentaries.* By Herbert Mayo, Surgeon, and Lecturer on Anatomy. 8vo. pp. 120; with Plates. T. and G. Underwood, London, 1822.

This little volume consists of distinct essays on various subjects, which the author purposes to continue at indefinite periods, and which, from our desire to give the earliest information on the present discussion about the nerves, we would have noticed in our last Number, had our limits admitted. The first essay is "on a Vital Principle;" and we were not at all sorry to find it very short, having an inherent antipathy to these unsatisfactory discussions. Mr. Mayo is inclined to believe that no change whatever takes place in the mental functions, except in conjunction with corresponding changes in the nervous system; but that mind and matter are logically distinct.

The second chapter, or division, consists in a detail of some experiments illustrating the phenomena of Muscular Action. In order to determine whether a muscle gain or lose in bulk during its contraction, a dog was killed by hanging, and the ventricular part of the heart introduced into a glass filled with coloured fluid and inverted, having a narrow tube, open, and three-tenths of an inch in diameter at its upper part, and a large stopper beneath. The vessel was raised so as to bring the tubular part uppermost; and, although the heart contracted vigorously, no elevation or depression of the fluid in the tube took place. The experiment, though not new in principle, seems well contrived and satisfactory. The author thinks that the habitual state of contraction of certain muscles, commonly called their *tone*, results from an act of volition; in illustration of which it is said, that, when all the branches of nerves passing to the lips of an ass are divided on either side, the lips hang flaccid; and that, in the human subject, when one side of the face is paralyzed, the features are partly drawn to the opposite side, and partly hang down merely from their weight. We do not, however, see the force of this example, which, in our judgment, only shows that the state of muscle called tone is preserved by nervous agency of some kind, but not that it is commanded by the will.

The next part of the work consists of four essays, by REIL, on the Anatomy of the Cerebellum, with eight engravings in illustration. The author has commenced his translations with the essays in the Archives of Physiology for 1807-8, but Reil had previously published a paper in Gren's Journal for 1795, in which he enters minutely into the structure of the brain, demonstrating its fibrous nature, and by a process similar to that which in this country has been supposed peculiar to Drs. GALL and SPURZHEIM. He says, "The structure of the medulla (or white substance,) is laminated, the laminæ are fibrous, and the fibres all radiate towards the surface. By a particular manipulation, the medulla may be separated into laminæ; yet not so easily, nor with so much regularity, as in the cerebellum; for in this last the laminæ are even and plain, while in the brain proper they are all curved and bent," &c. The plan of *scraping* seems to have been first practised by VIEUSSENS, and the fibrous structure demonstrated; and, about twenty years after him, the subject was taken up by Reil, whose first essay we have said was published in 1795, and ten separate dissertations are to be found in a journal edited by himself, (Archiv für die Physiologie,) between the years 1807 and 1812. To these Mr. Mayo has now directed the attention of his professional brethren, stating them to be nearly unknown in this country. From this general imputation of neglect to our anatomists, which we believe to be correct, we must except the late Dr. JOHN GORDON, of Edinburgh, who was in the habit of demonstrating the brain from preparations made after the manner of Reil, to whom, we believe, much of the credit is due for discoveries which have been given to his countrymen, Gall and Spurzheim. The method of Reil is thus described with regard to the cerebellum:

"The cerebellum of a male should be selected, and of one who may have died in early manhood of some chronic disease; it should be in as fresh a state as possible: the brains of those who have died of typhus lose their consistence too soon for this purpose, and, where inflammation of the brain has existed, the membranes are not easily separable. The cerebellum may be detached by dividing the crura cerebri above, and the medulla oblongata below; it should then be placed in a basin under water, and the membranes removed with the forceps: the membranes are prevented from drying, and the blood exudes more freely, when the part is thus immersed in water. The denuded cerebellum is now to be placed in a vessel, and to be twice washed by the affusion of brandy, which may be suffered to remain on it some minutes; afterwards alcohol is to be substituted, in which it should stand twelve hours. When in this way the surface appears somewhat hardened, the membrane is to be removed from the deeper furrows, in order that the spirit may every where penetrate the mass; spirit is then again to be poured over the preparation, which may stand a day or two. Finally, the alcohol is to be renewed, and the vessel closed and set by for two or three months, till the part has acquired a greyish colour, and is thoroughly hardened. It is right, during this time, to turn the preparation occasionally, and to contrive that every surface is freely bathed in the spirit."

The subject is discussed with an extreme degree of minuteness, which renders these essays of great value to anatomists, on whom Mr. Mayo has conferred an essential obligation by rendering them thus easily accessible, and we trust he will be induced to continue his translations.

The concluding part of the volume is devoted to a question which is likely to prove of great interest,—viz. the functions of certain nerves, in reference to the doctrines of Mr. Bell and Mr. Shaw. We do not mean at present to enter into any formal discussion of the subject, but to give the evidence on either side, as it may from time to time appear, and have now to lay before our readers some experiments and observations by Mr. Mayo, intended to invalidate the opinions of Mr. Bell.

"*Experiment* 1.—The infraorbital and inferior maxillary branches of the fifth were divided on either side, where they emerge from their respective canals: the lips did not lose their tone, or customary apposition to each other and to the teeth; but their sensibility seemed destroyed. When oats were offered it, the animal pressed its lips against the vessel which contained the food, and finally raised the latter with its tongue and teeth. On pinching with the forceps the extremities nearest the lips of the divided nerves, no movement whatever of the lips ensued. On pinching the opposite extremities of the nerves, I observed that the animal struggled violently, as at the moment of dividing the nerves: these latter results uniformly attend the division of the nerves above mentioned, and of that branch of the fifth which joins the portio dura. Some days afterwards, though the animal did not raise its food with its lips, the latter seemed to be moved during mastication by their own muscles.

"*Experiment* 2.—The common trunk, composed of the portio dura and a branch of the third division of the fifth, was divided upon the masseter muscle on either side: the lips immediately fell away from the teeth, and hung flaccid, and the nostrils lost all movement. The sensibility of the lips appeared unimpaired; the animal raised its food as in the former instance. When the extremity nearest the lips of either divided nerve was pinched, the muscles of the lips and nostril on that side were convulsed.

"Some days after this, the frontal nerve was divided on one side of the forehead of the same ass; when the neighbouring surface appeared to have lost sensation, but its muscles were not paralyzed.

"*Experiment* 3.—The portio dura was divided on either side immediately before its union with the branch of the fifth pair: the muscles of the lips and nostrils seemed as thoroughly paralyzed as in the preceding experiment.

"*Experiment* 4.—That branch of the fifth which joins the portio dura was divided on either side: at first the under lip appeared to fall away from the teeth, but not to the same degree as in the two former instances; at times the lips were justly closed; and the animal invariably raised with its lips, as readily as before the division of the nerves, the oats which were at intervals offered it. The asses employed in these experiments, with the exception of the first two, were killed as soon as the effect of the operation had been satisfactorily ascertained, in order to determine by dissection whether the division of the nerves had been completely effected: in this instance it was found that, on one side, a fine filament, of the size of a common thread, passed from the branch of the fifth to the portio dura, before the place of the division of the former. No difference had been observed between the action of the muscles on either side of the face.

"*Experiment* 5.—A repetition of the preceding; but on one side a larger filament had been left undivided. In this case the under lip did not hang down; no difference had been noticed between the action of the muscles of either side.

"*Experiment* 6.—A repetition of the preceding, with exactly the same result as in Experiment 5th; and still on one side a filament of the size of a thread had been left, uniting the fifth with the seventh. Upon the same animal, the infraorbital and submaxillary nerves were divided: the upper lip was now observed to hang down a little on each side; but this circumstance seemed fairly attributable to the very extensive division of the muscular fibre on that side. The portio dura was finally divided on one side, where it emerges from the skull: the animal was observed to lose instantly the power of closing the eyelids on that side; to determine which point alone the division of the 7th, near the skull, had been intended."

The inference drawn by Mr. Mayo from these experiments is, that the portia dura in the ass is a "simple nerve of voluntary motion;" while the frontal, infraorbitary, and inferior maxillary branches, are "nerves of sensation only, to which that branch of the fifth which joins the portia dura probably contributes;" and that other branches of the third division of the fifth pair are voluntary nerves to some of the neighbouring muscles. The author endeavours to show that the nerves which Mr. Bell calls respiratory do not differ in any important circumstance, as a class, from those with which he contrasts them. His reasoning is as follows.

"1. The par vagum: this nerve has many roots, and has a ganglion near its origin. When the branches of the par vagum which pass to the larynx are divided, the voluntary movements of that organ are destroyed; the part is no longer competent to the formation of sounds, or to assist in the act of deglutition; while, on the other hand, respiration is not impeded. The par vagum is acutely sensible: I exposed its trunk in the neck of an ass, and, on pinching it with the forceps, the animal gave violent indications of pain.

"2. The portio dura of the seventh is proved, by the experiments which I have detailed, to be a common nerve of voluntary motion: if it be divided, the muscles which receive branches from it are completely paralyzed.

"3. The spinal accessory nerve: of this Mr. Bell observes, that 'it controls the muscles of the neck and shoulder in their office as respiratory muscles, when, by lifting the shoulders, they take the load from the chest, and give freedom to the expansion of the thorax. When it is cut across in experiments, the muscles of the shoulder, which were in action as respiratory muscles, cease their co-operation, but remain capable of voluntary actions.'

"In human beings, the only muscles of the neck and shoulder which receive branches from the spinal accessory, are the sterno-cleido-mastoideus and the trapezius.

"4. The phrenic nerve is formed of branches of four or five spinal nerves; it generally receives a fine filament or two, from the ninth pair, the par vagum, and the sympathetic.

"5. The posterior thoracic nerve is formed of branches of the spinal nerves."

The author next proceeds to state objections to the experiments of Mr. Bell, (see Philosophical Transactions, vol. cxi.) In the first of these the portio dura was divided, in an ass, upon one side of the head: "the motion of the nostril of the same side instantly ceased, while the other nostril continued to expand and contract in unison with the motions of the chest." Mr. Mayo objects to this experiment as inconclusive, because the nerve was not divided on both sides, and thinks that, if it had been so, a different result would probably have ensued. Next an ass had the superior maxillary branch of the fifth pair divided: no change took place in the motion of the nostril, "but the side of the lip was observed to hang low, and it was dragged to the other side." The opposite branch of the fifth pair was then divided in the same animal: and it was now found that "the power of elevating and projecting the lip, as in gathering food, was lost." In this abridged account of the experiment, it will be observed that two clauses are marked as quotations: to the former, Mr. Mayo objects that it is contrary to his own observation; to the latter, that it is but a theoretical account of the fact, that the animal did not elevate and project the lip,—a fact which he thinks is proved, by his third experiment, to arise merely from the part having lost its sensibility. Again, Mr. Bell states that, in creatures which do not breathe, the mouth, having but one function to perform, has but one nerve. Mr. Mayo says, that the ass does not appear to breathe through its mouth, yet the portio dura in this animal sends branches to the lips, the division of which, and of which alone, paralyzes the muscles of the lips.

We believe we have laid before our readers the principal evidence and reasoning brought by Mr. Mayo against the Windmill-street doctrine; and, although they evince considerable ingenuity, we do not think them so irrefragable as to warrant the conclusion which he gives in the following terms:

"It remains for the reader to decide, whether Mr. Bell's experiments are satisfactory, and bear out his inferences; whether the latter, coupled with my former observations on the five 'respiratory nerves' of this author, leave his theory tenable; and, perhaps, finally to determine whether there exist in the whole of Mr. Bell's essay, after the deduction of his controvertible statements, more than one correct inference. I here allude to Mr. Bell's experimental confirmation of an opinion which, at the beginning of the eighteenth century, occurred to Dr. Blair, on his minute examination of the proboscis of an elephant,—viz. that the infraorbital nerves are nerves of touch."

The question at issue is one of great interest; and we have only at present to add, that we trust the discussion will be conducted, on either side, with that spirit of moderation and candour which ought to influence members of a liberal profession, emulous in the improvement of science and the pursuit of discovery.

Art. II.—*A Treatise on Animal Magnetism.* By M. Loewf, Doctor of Medicine, Philosophy, and Surgery, &c. Schulze, London, 1822.

Most of our readers are aware that the science of animal magnetism has lately been very much cultivated in Germany, where many men of talent have investigated the subject, and been led to embrace its doctrines. In this country it has been always treated with contempt, and few among us think of viewing the science in any other light than that of quackery and imposition.

If any thing were necessary to prevent these opinions from gaining ground in this country, it would be the essay before us, by M. Loewe, a doctor of philosophy, surgery, and medicine, who, although from the land where animal magnetism is in vogue, and where many tolerable papers on that subject have been published, gives but an indifferent account of the science, in favour of which he meant to prepossess us. Indeed, had it not been for the title-page, we could not have discovered what was the nature of the subject under discussion. A few extracts will best illustrate the manner of our author, and, perhaps, account for the difficulty we have experienced in comprehending his speculations.

Speaking of Identity, he observes, "that the *pure ideal* and the *pure real* are merely different views of one and the same *unity*, and are consequently identical; and that, in consequence, the *a priori* and the *a posteriori* must also be identical."

On Life and Death he remarks, "according to our conception, absolute death does not exist. That which we in general call death is nothing else but an alteration of form, affected by means of a modification of life."

After having waded through numberless unintelligible paragraphs on life and death, we come to the following summing-up, the meaning of which we leave as a task for the ingenuity of our readers to discover:—"The result of all this is, that matter and spirit, being and form, subject and object, denote only identical notions."

To show the difference between natural and morbid sleep, our author tells us the two following very amusing stories:

"A Prussian officer, in a battle during the seven years' war, just in the moment as he was going to cry out 'Bravo, comrades!' received a blow on the head, and fell senseless to the ground. Some of the enemy found him lying there, and, supposing him to be dead, plundered, and left him quite naked in a ditch. Some peasants, who found him in that state, took him home, and he recovered. However, he had entirely lost the power of recollection and his speech, and could not even tell his own name. He roved about the country for a considerable time, and at length he was taken up by the police as a vagrant, and was conveyed to a country gaol: here he was obliged to work at the fortifications of the town, and his dumbness was supposed to be pretended. One day he, together with his wheel-barrow, fell down the ramparts, but scarcely had he reached the ground when he bawled out, 'Bravo, comrades!' The task-masters, as well as the rest of the prisoners, viewed him with surprise; and he himself was not less astonished at his condition, nor could he possibly conceive how he came to this place. He told his name, but they laughed at him: however, at his own request, he was brought before the commandant, and, after a strict investigation, he obtained his liberty in the most honourable manner.

"A boy, employed to put up nine pins, was hit on the head by the ball, which had thrown down all the pins, just at the moment when he was going to cry out 'All nine!' He fell senseless to the ground,—after a while got well again,—but remained for several years without the use of all his higher faculties, and without all power of recollection. One day he fell down a flight of stairs, and suddenly cried out 'All nine!' and from that moment he regained his perfect senses. But neither of these persons subsequently recollected a word of what had happened to them during the interval."

It is matter of much regret that the author does not give his authority for these anecdotes; as he informs us, that he was unable to remember whether he had read them in the works of the celebrated REIL, "or in a similar work," or, lastly, whether he had not heard them from Professor KIESEWETTER, at Berlin.

Our author proceeds to tell us that some persons, who have been magnetized, become possessed of the faculty of *clairevoyance*, or what the Scotch call the *second sight*; and, when asked in what manner they are sensible of these impressions, they reply, "that they are not able rightly to describe it, but say it seemed as if somebody whispered it to them." Persons magnetized also know the thoughts of others, "particularly as the *real* and *ideal* states are identical." It is to be observed, that the more healthy a person becomes, the less susceptible he is of the magnetic influence.

In the concluding part of his treatise, the author gives a few cases as examples of the efficacy of animal magnetism in the cure of diseases: two of these we shall lay before our readers, having first warned them that the remedy is declared by the author to be fraught with danger in the hands of ignorant practitioners.

"A lady, residing in London, after a violent *febris nervosa putrida*, under which she was suffering in the months of December and January last, was affected by convulsions of every kind, but mostly by fainting, which often lasted two hours, and it was difficult to bring her to herself. I was one day present when this fainting was coming on, and, in presence of her sister and brother-in-law, I tried to make application of animal magnetism. I had scarcely begun to operate, when she quickly recovered from the fainting, as though she had been awakened by fright, and from that moment she gradually recovered, *with the assistance of medicaments usual in such cases*, and is now in perfect health.

"A gentleman, thirty-five years of age, had laboured for many years under a chronic disease, which sometimes represented itself in the form of head-ach, particularly of one side of the head, (*hemicranium*,) and sometimes as an hypochondriac affection: after having made use of all the medicines usual in such diseases without effect, he was magnetized. For three weeks, during which time the animal magnetism was daily repeated, there was no remarkable difference in his case; but, in the fourth week, the symptoms of the first degree of magnetism presented themselves, and he was brought to the state of sleeping, and, after the daily repetition of animal magnetism during the space of *eight weeks, he recovered without any of the phenomena taking place*."

He concludes with informing us, that animal magnetism, if properly applied, would be useful in suspended animation. "It is well known," says he, "that, in the case of drowned persons, one of the most effectual remedies is to place the patient in bed between two naked persons. Such a remedy has been resorted to, under my directions, with complete success."—A hint to the Humane Society.

His description of a person magnetized is the following:—" Quietude and cheerfulness, and an universal sereneness, is spread over and depicted on the countenance. They enter, in a new mode, into a relation with the external world by entirely different organs, and, in particular, are dependent on their magnetiser, so that they can perceive only by him. They recognize only those persons who are in the same *report* with him as themselves. In this shutting-up of the external senses, the internal sense receives, with uncommon clearness, the influence of subjects by unusual parts, and particularly by the region of the stomach, by the tips of the fingers, by the forehead, nay, even by the crown of the head. In this manner they can often read closed letters."

We have neither limits nor inclination to dwell longer on this ebullition of German enthusiasm and folly; and indeed we might not have noticed it at all, but for the exhortation of the author to professional men " to clear the subject from all the impurities and absurdities" with which it has been mixed; and we find, further, on the authority of Dr. Loewe, that illegal practitioners have been " emboldened in their audacities from neglect."

